# ‘Unheard voices’: Perceptions of women with mental illness on nurses screening routinely for domestic violence: A qualitative analysis

**DOI:** 10.17533/udea.iee.v39n3e03

**Published:** 2021-11-05

**Authors:** Vijayalakshmi Poreddi, S. Sai Nikhil Reddy, Sailaxmi Gandhi, Marimuthu P, Suresh BadaMath

**Affiliations:** 1 Ph.D. Clinical instructor, College of Nursing. National Institute of Mental Health and Neuro Sciences Bangalore, India Email: pvijayalakshmireddy@gmail.com. Corresponding author National Institute Of Mental Health And Neurosciences National Institute of Mental Health and Neuro Sciences Bangalore India pvijayalakshmireddy@gmail.com; 2 MBBS. Bangalore Medical College and Research Institute, Bangalore, India. Email: saithereddy@gmail.com Bangalore Medical College and Research Institute Bangalore India saithereddy@gmail.com; 3 Ph.D. Professor, Department of Nursing. National Institute of Mental Health and Neuro Sciences Bangalore, India Email: sailaxmi63@yahoo.com National Institute Of Mental Health And Neurosciences National Institute of Mental Health and Neuro Sciences Bangalore India sailaxmi63@yahoo.com; 4 Ph.D. Professor, Department of Bio-statistics. National Institute of Mental Health and Neuro Sciences Bangalore, India Email: p_marimuthu@hotmail.com National Institute Of Mental Health And Neurosciences National Institute of Mental Health and Neuro Sciences Bangalore India p_marimuthu@hotmail.com; 5 MD in Psychiatry, Professor, Department of Psychiatry. National Institute of Mental Health and Neuro Sciences Bangalore, India Email: nimhans@gmail.com National Institute Of Mental Health And Neurosciences National Institute of Mental Health and Neuro Sciences Bangalore India nimhans@gmail.com

**Keywords:** battered women, mental disorders, physical abuse, qualitative research., mujeres maltratadas, trastornos mentales, abuso físico, investigación cualitativa., mulheres maltratadas, transtornos mentais, abuso físico, pesquisa qualitativa.

## Abstract

**Objective:**

To explore women's experiences of violence and their opinion on routine screening for domestic violence by nursing professionals in mental health care settings.

**Methods:**

This qualitative narrative research design was carried out among 20 asymptomatic women with mental illness at a tertiary care centre in Bangalore, India.

**Results:**

Narrative content analysis was performed, and five dominant themes have emerged: 1. Understanding the nature and signs of violence (subtheme: Meaning of violence), 2. Abusive experiences of women with mental illness (subthemes: Physical violence, psychological violence, social violence, sexual violence and financial violence), 3. Experiences on disclosure of violence (subthemes: Identification of violence by nursing professionals, Experiences of disclosure of violence), 4. Barriers for disclosure of abuse(subthemes: Fear of consequences, the hectic schedule of nursing staff, helplessness and hopelessness, perceived poor family support). 5.Routine screening for violence by nursing professionals (subthemes: reasons for routine inquiry of violence, nature of inquiry by the nursing professionals).

**Conclusion:**

Women with mental illness were undergoing more than one form of violence, and most of the participants supported routine screening by nursing professionals. Nurses play an essential role in identifying and supporting abused women in mental health care settings**.**

## Resumen

**Objetivo:**

Explorar las experiencias de violencia que sufren las mujeres y su opinión sobre el cribado rutinario de la violencia doméstica por parte de los profesionales de enfermería en los centros de salud mental.

**Métodos:**

Esta investigación narrativa cualitativa se llevó a cabo con 20 mujeres asintomáticas con enfermedades mentales en un centro de atención terciaria en Bangalore, India.

**Resultados:**

Se realizó un análisis de contenido narrativo y surgieron cinco temas dominantes: 1. Comprensión de la naturaleza y los signos de la violencia (subtema: Significado de la violencia), 2. Experiencias abusivas de las mujeres con enfermedades mentales (subtemas: Violencia física, violencia psicológica, violencia social, violencia sexual y violencia económica), 3. Experiencias sobre la revelación de la violencia (subtemas: Identificación de la violencia por parte de los profesionales de enfermería, Experiencias de revelación de la violencia), 4. Barreras para la revelación del abuso (subtemas: Miedo a las consecuencias, el agitado horario del personal de enfermería, impotencia y desesperanza, percepción de un escaso apoyo familiar). 5. Indagación rutinaria de la violencia por parte de los profesionales de enfermería (subtemas: razones para la indagación rutinaria de la violencia, naturaleza de la indagación por parte de los profesionales de enfermería).

**Conclusión:**

Las mujeres con enfermedades mentales sufrieron más de una forma de violencia y la mayoría de las participantes apoyó el cribado rutinario por parte de los profesionales de enfermería. Las enfermeras desempeñan un papel esencial en la identificación y en el apoyo a las mujeres maltratadas en los entornos de atención en la salud mental.

## Resumo

**Objetivo:**

Explorar as experiências de violência sofrida por mulheres e sua opinião sobre o rastreamento rotineiro de violência doméstica por profissionais de enfermagem em centros de saúde mental.

**Métodos:**

Esta pesquisa narrativa qualitativa foi realizada com 20 mulheres assintomáticas com doença mental em um estabelecimento de cuidados terciários em Bangalore, Índia.

**Resultados:**

Realizou-se uma análise de conteúdo narrativo e emergiram cinco temas dominantes: 1. Compreendendo a natureza e os sinais da violência (subtópico: Significado da violência), 2. Experiências abusivas de mulheres com transtorno mental (subtópicos: Violência física, violência psicológica, violência social, violência sexual e violência econômica), 3. Experiências sobre a divulgação da violência (subtópicos: Identificação da violência por profissionais de enfermagem, Experiências da divulgação da violência), 4. Barreiras para a divulgação do abuso (subtópicos: medo das consequências, enfermagem ocupada horas de trabalho, desamparo e desesperança, percepção de pouco apoio familiar) 5. Inquérito de rotina sobre violência por profissionais de enfermagem (subtópicos: motivos de inquérito de rotina sobre violência, natureza do inquérito por profissionais de enfermagem).

**Conclusão:**

Mulheres com doença mental sofreram mais de uma forma de violência e a maioria das participantes apoiava o rastreamento de rotina pelos profissionais de enfermagem. Os enfermeiros desempenham um papel essencial na identificação e apoio às mulheres agredidas em ambientes de cuidados de saúde mental.

## Introduction

Violence against women is a global public health issue. Violence among women is defined as “an act of gender-based violence that results in physical, sexual, or psychological harm to women including threats of such acts, coercion or arbitrary deprivation of liberty, whether occurring in public or private life”. (1) According to the National Family Health Survey (NFHS-4, 2015-16), 27 percent of women have experienced physical violence since the age of 15 in India. (2) A systematic review showed that 41% of Indian women experienced domestic violence (at least two forms of abuse) during their lifetime and 30% in the past year.(3)

International studies have established a clear association between violence against women and poor mental health.(4) Furthermore, systematic reviews have reported that people with mental illness are at increased risk for violence(5,6) than the general population.(7) The lifetime prevalence of domestic violence among psychiatric in-patients ranged from 30% to 60%, with a higher prevalence in women compared to men. Further, a recent nationwide survey from the UK found that around one in five women with severe mental illness (SMI) experienced domestic violence, and one in ten women (and 3% of men) with SMI experienced sexual violence.(8) However, their contact with mental health services provides a window of opportunity for mental health professionals to identify and provide appropriate interventions. In India, women who had experienced domestic violence were more likely to report poor mental health and suicidal tendencies compared to women who had not experienced violence.

World Health Organization recognized violence against women as a health priority and declared on strengthening the role of the health system in addressing violence against women.(9) While people with mental illness are at greater risk for victimization,(10) often goes unnoticed by mental health professionals. There has been limited research that explored views of women with mental illness on an understanding of abuse, barriers to disclosure of violence and opinion on routine enquiry by mental health professionals.(11) A handful of Indian studies have examined the prevalence of violence among persons with mental illness(12) and very few qualitative studies have explored violence among women with mental illness.(13) Qualitative research is an appropriate approach in exploring the abusive experiences of women with mental illness because of the complex nature of the mental illness. The present study aimed to explore experiences of violence among women with mental illness and their opinion on routine screening for domestic violence by nursing professionals in mental health care settings.

## Methods

### Research setting and participants

This study involves a qualitative research method using narrative inquiry to understand women with mental illness views about the routine enquiry by the nursing professionals about domestic violence at a tertiary care centre, Bangalore, India. The study criteria include female psychiatric patients who were asymptomatic (preferably before discharge) and have a history of domestic violence experience. Symptomatic and intellectually disabled patients were excluded from this study. The primary author approached the concerned resident doctor to discuss the severity of the symptoms and gone through the case files to confirm abusive experiences among the participants. The participants were recruited purposively to ensure diversity regarding age, diagnosis, religion, marital status, background, number of hospitalizations, and diagnosis. We determined that theoretical saturation was achieved after 20 interviews (out of a total of 24) as analysis of the four subsequent interviews did not generate any further leads. The mean age of the participants was 30.17 years (range 22-45, SD, 6.30). Sixteen were Hindus, three were Christian and one participant belonged to the Muslim religion. More participants were from rural backgrounds (*n*=14), and six were from an urban background. Only two of the participants were employed, while a majority were homemakers (*n*=18). The diagnoses of the participants as follows; depression (*n*=9), Schizophrenia (*n*=4), BPAD (*n*=5), psychosis(*n*=2). Most of the participants were hospitalized for the first time (n=14), married (n=16), and had children. However, three of the participants were deserted by their husbands.

This study used a modified Interview guide (11 semi-structured questions) initially developed by Rose et al. (2011).(14) The researchers obtained the necessary permission from the authors to adapt and make modifications to meet the objectives of the present study. The following are examples of questions asked in interviews: ‘Can you describe the kind of violence you are experiencing by family members” “Have you ever been asked about abuse by nursing professionals? if yes, what was your experience of this? How did it make you feel? Nature of the enquiry, ‘Do you think that nurses should ask all clients about abuse? If not, why not? If yes, why? Do you think nurses should ask women with mental illness if they have ever been abused?

The modified interview guide was given to mental health experts from various departments (Psychiatric Nursing, Psychiatry, Psychiatric Social work, Clinical Psychology and Biostatistics) and incorporated the suggestions given by the experts. The finalized interview guide was piloted among five female psychiatric inpatients and found it was feasible to adapt to Indian settings. Since the participants were the vulnerable group, the primary author met concerned nurses and requested them to enquire about their willingness to participate in the study. Then the researcher met the potential participants, briefly explained the study's aims and objectives, and obtained the written informed consent. After developing a good rapport, the primary author individually interviewed the participants in interview rooms at psychiatry units. The interviews were conducted between January and May 2017. Each interview took approximately 45 minutes to 60 minutes which were then audiotaped and transcribed verbatim.

### Ethical considerations

The study received ethical approval from the Institute Ethical Committee (NIMH/DO/IEC (BEH. Sc. DIV)/2016). After explaining the aims and objectives of the study briefly, the participants provided written informed consent for their participation and audio recording. Though we have included asymptomatic patients in this study, the authors consulted the treating doctors and obtained their approval to conduct the interviews since the participants were the vulnerable group. They were also informed that they were given the freedom to withdraw from the study at any point of time without giving any reason. The researchers have maintained anonymity and confidentiality as they conducted the interviews in a private room in the hospital, and participants were given code numbers for analysis purposes. Also, the researchers removed the participant names and other personal identifiers from the transcripts. However, the interviews conducted by the primary author (PV) ‘who said what’ was not divulged. The researcher is the only person aware of the codes and did not share the information with anyone, including the research supervisor. Brief counselling was provided by the primary author (PV) if any significant distress occurs among the participants during the interview and offered appropriate guidance.

### Data analysis

Data analysis involved thematic analysis and was conducted concurrently with data collection. The data analysis and coding procedures followed the six steps suggested by Creswell.(15) The first step was the collection of descriptions based on the patients' experiences. In the second step, the primary author (PV) converted the verbatim transcription of the interviews (regional languages) into English text, and the supervisors (SG, SBM) randomly checked transcriptions for accuracy. This step also included rereading the transcribed text to gain a general understanding. In the third step, preliminary codes were developed after reading the transcripts for several times. However, the coding scheme was modified as new interviews were coded. Codes were organized using Atlas ti software (version 7.5.10). the researchers categorized the related concepts. In the fourth step, the organized concepts were described and examined in more detail. Codes were built into broader categories through constant comparison and provisional themes were developed. The researcher interlaced emergent themes from the participants' responses into a brief description in the fifth step. The final step included the interpretation of data.

### Rigor

The data was validated using the Lincoln and Cuba criteria (credibility, transferability, dependability, and confirmability). The participants were requested to offer their feedback on transcripts for the accuracy of the paraphrasing, and one participant provided comments and corrections. The credibility of coding is maintained by involving two researchers (PV& SNR) to code the same transcript individually and then discuss any similarities and differences in the resulting sets of codes supervised by the research guides (SG, MP). For transferability, the researchers used thick descriptions to describe the experiences of the participants with maximum variation. Two qualitative research experts checked the emerged themes and subthemes for dependability and confirmability.

## Results

The data was saturated with a purposive sample of 20 female psychiatric patients. Findings are presented in response to the questions. In this study, five themes and fourteen subthemes have emerged (Table 1).

**Table 1:** Derived Themes and subthemes

Themes	Subthemes
1. Understanding the nature and signs of violence	1. Meaning of violence
2. Abusive experiences of women with mental illness	1. Physical violence
	2. Psychological violence
	3. Social violence
	4. Sexual violence
	5. Financial violence
3. Women’s’ experiences on disclosure of violence	1. Identification of violence by nursing professionals
	2. Experiences of disclosure of violence
4. Barriers for disclosure of violence	1. Fear of consequences
	2. Hectic schedule of nursing staff
	3. Helplessness and hopelessness
	4. Perceived poor family support.
5. Women’s views on routine screening for violence by nursing professionals	1. Reasons for routine inquiry of violence
	2. Nature of inquiry by the nursing professionals

### Theme 1. Understanding the nature and signs of violence

The researchers in this study explored the participants' understanding of the nature and signs of violence.

*Subtheme1. Meaning of violence.* In this study, all the participants were aware of the meaning and types of violence, such as physical violence, sexual violence, and psychological violence. However, few of them were unaware of social and financial violence. Few of the responses from the participants include: *I think violence means …. Beating …. Pushing the women in an inhuman way (X5); Behaving differently to entrap women and torturing them (X6); Violence usually occurs by men to the women … I mean making her cry… (X8); Scolding women for simple things…. Purposefully making her cry (X9).*

### Theme 2. Abusive experiences of women with mental illness

Most of the participants agreed that they were the victims of physical, psychological, and sexual violence. While they were not aware of financial and social violence, they could reveal their experiences after probing.

*Subtheme 1. Physical violence.* In this study, all the participants expressed that they have experienced physical violence by family members. A few participants said that their family members also threatened to admit them to the psychiatric hospital as they burden the family. Few of the narratives include: *See this scar… I had an injury when my husband pushed me against the wall…. He says I am useless…. If I irritate him, he will put me in the hospital…. (X3); My brother hit me in the bus stand ….in front of everybody… I felt very sad… I reported to my mother … she too supported my brother. She treats me low … even before I have developed this illness… she controls me. She doesn’t give me money… I have to eat whatever is left over after my brother having…. Both of them say that I am good for nothing … (X4).*

*Subtheme 2. Psychological violence.* Similar to physical violence, all the participants stated that they had undergone psychological violence. Further, they expressed that physical scar can be disappear gradually, but the psychological trauma makes them feel isolated and sad. Few of the participants thought that they were treated differently by their family members. Two of the participants revealed that their husbands remarried without divorcing them. But unfortunately, their husbands were supported by the family members. Examples of psychological violence include: *Yes, my family members treat me differently…. They show anger unnecessarily… (X1); My husband left me at my parents’ home after I have developed this illness... he married again with my cousin…. My parents also supported this…. I felt very hurt… what is my fault……. (X5).* While it is important to consider the fact that the types of violence are interrelated, as the same individual undergoes physical and psychological violence……. As one of the participants expressed that Physical injuries can heal …but the psychological hurt …. And those feelings are still raw in my mind as I have undergone abortion because my husband pushed when I was five months pregnant and never conceived again…. (X8).

*Subtheme 3. Social violence.* Most of the participants in this study were aware of social violence as they expressed that they were not allowed to attend family gatherings and continue their education. One of the participants said that their family members are forcing her to marry her uncle. Few narratives of social violence include: *My husband not taking me out even for my relatives’ functions... he says I look like pig… he also says he is ashamed of me… I don’t know how to behave…. (X4); My family members stopped me going to college... they say I cannot achieve anything… they are forcing me to get married to my uncle… I don’t like him… he misbehaved with me….. (X2).*

*Subtheme 4. Sexual violence.* Few of the participants in this study have expressed that they experienced sexual violence by family members. One of the participants clearly explained the impact of sexual violence on her and her sister as they got admitted to the psychiatric hospital. Her story reflects the way few men take advantage of helplessness in women: *My husband is an alcoholic… my sister stays with me… because our parents passed away five years back... My sister was alone at home, and my paternal uncle raped her…. After that incident, my sister was admitted to a psychiatric hospital with severe depression... After discharge, I have taken her to my house…. two weeks back …. Again, she was sexually abused by my husband…. When I questioned him … he says that he has done good …for her. She will improve now. After this incident, my sister attempted suicide, and I readmitted her…. Doctors said that I too need treatment, and now I am on medication… I have two small children at home… I don’t feel like to go home and to stay with him……. (X8).*

*Subtheme 5. Financial violence.* After probing, a few of the participants revealed to their experiences of violence related financial matters. They also felt that psychological and physical violence traumatize them more than financial violence. Few examples of financial violence include; *My husband doesn’t involve me in any financial matters…. I am the one who used to manage the home…now my husband believes his mother…. I feel sad … (X6).* All the participants agreed that they are experiencing various types of violence, making them feel sad, helpless, and need someone to understand them. The above illustrations provide a piece of clear evidence that most of the participants are experiencing more than one type of abuse.

### Theme 3. Women’s’ experiences on disclosure of violence

The participants reported their experiences of nurses identifying violence and their responses to the disclosure of violence experiences.

*Subtheme 1. Identification of violence by nursing professionals.* All the participants in this study felt that nurses are friendly and approachable. Yet, nurses did not inquire them about violent experiences during their stay in the hospital or during the discharge. Few of the participants expressed that they were enquired about violence experiences when they see physical scars on their bodies and if they were moody and any changes in their behaviours such as not participating in the unit activities or refusing to take medicines or changes in eating or sleeping. Few of the narratives include: *Yes, I remember... One of the sisters asked me when she has seen this scar…. (X3); Yeah…. all the sisters are nice….Friendly… but none of them asked about abuse (X5); No one asked me about this …. I tried telling them but … I felt … they won’t believe me (X8).*

*Subtheme 2. Experiences of disclosure of violence to the nursing professionals.* Few of the participants stated that they have disclosed their experiences of violence with the nursing professionals and felt a feeling of relief. Few of the participants said they wanted to reveal their incidents of violence, but the nurses were either not interested in listening or didn’t respond: *My brother and my mother hit me in the ward and front of nurses…. No one interfered…. Later, the sister came and counselled my mother…. (X6); One of the nurses asked me since I did not have food …. She asked me about this… I have told all of my problems…. I felt relieved… (X7).* While most of the participants expressed that disclosing violence to the nurses helped them in ventilating feelings, two participants felt that it may not help them improve their situation so they didn’t disclose to the nurses.

### Theme 4. Barriers for disclosure of violence

Participants in this study were encouraged to express the reasons for not disclosing abusive experiences with health care professionals. This main theme consisted of four subthemes: fear of consequences, hectic schedule of nursing staff, helplessness and hopelessness and perceived poor family support.

*Subtheme 1. Fear of consequences.* The majority of the participants wanted to disclose their experiences of violence with nurses. However, they had a fear that revealing of violence by their family members may be amplified. Few participants said they were worried that nurses might not believe their words or make fun of them. Few of the narratives include: *I feel like expressing about my husband’s behaviour … but I am terrified ... I don’t have any support… (X12); I felt bad… when I tried to disclose my experiences, the sister said all that because of my mental illness…They may make fun of me after listening to my experiences (X2); Nurses may reveal this to my husband because he may throw me out of the house (X4).*

*Subtheme 2. The hectic schedule of Nursing staff.* Few of the participants expressed that they wanted to share their violence experiences with the nursing professionals. But they did not disclose as nurses were very busy with their routine work: *I tried expressing with one of the nurses…. She said that she is swamped… (X14); To be frank ,I have disclosed my experiences… but the nursing staff did not respond and said that she will would talk to me later… (X9).*

*Subtheme 3. Helplessness and hopelessness.* Very few of the participants felt that disclosing their abusive experiences with nursing professionals may not help them find the solutions. One of the participants stated the following: *I don’t want to tell anyone because no one can help me….. (X15).*

*Subtheme 4. Perceived poor family support.* The majority of the participants believed that disclosing self-experiences of violence might impact their relationships with the family members. The family members do not support them in expressing their difficulties with health care professionals. Further, they require family members help in follow-up care and in meeting their basic needs. Few of the narratives include: *I am worried …. My husband may not accompany me to the hospital for the follow-up… he may not buy medications for me... (X12).*

### Theme 5. Women’s views on routine screening for violence by nursing professionals

Participants were encouraged to express their expectations from nurses when they enquire about sensitive issues such as violence experiences. This central them consisted of two sub themes: reasons for routine inquiry of violence and nature of inquiry by the nursing professionals.

*Subtheme 1. Reasons for routine inquiry of violence by the nursing professionals.* All participants in this study felt that nurses are friendly, approachable, accessible at all times, and support them in finding solutions for their problems. Below given are few reasons endorsed by the participants. Most participants opined that they are comfortable disclosing their abusive experiences with the nurses as they are the most trusted individuals. However, most of them felt that routine inquiry by nurses helps them disclose violence experiences without any discrimination. Few of the narratives include: *Nurses are friendly, and I have no fear that they may reveal to family members… by expressing with them, I feel a bit relaxed (X10); Nurses may find the solutions… they may help us …. (X12); Unless nurses ask about violence, many of us are not aware whether we can ventilate about our abusive experiences or not … (X2); Nurses may speak to elders in our family to find solutions to stop violence among us (X1); I feel guilty to express, but the sister spoke to me in such a way… I have told my story… she referred me to the social worker... Now I am waiting to go as per their suggestions I can’t go back to my husband with my sister who is mentally disturbed than me… (X4); If nurses ask routinely to all the patients.... I won’t feel the nurses discriminate me…. I may not worry that they are documenting this violence… (X7).*

*Subtheme 2. Nature of inquiry by the nursing professionals.* In this study, most participants felt that nurses should inquire about violence in a polite manner. They should listen to them patiently, confidentiality assured, and above all, nurses should believe their violence experiences. The following narratives explain the participants' expectations in enquiring about the experience of violence among women with mental illness: *I feel nurses should universally inquire about violence to all women patients…. It may help them to open up and ventilate their feelings. But they have to believe and listen to us patiently... (X2, X4, X5); I wanted them to ask politely……. they should listen to me what I am saying…. (X1,3); I want nurses to respect me… should believe me and should not inform to the family members (X11).*

*Nurse should understand my situation empathetically… I don’t want them to sympathize with me (X13).*

## Discussion

This was the first study that explored the abusive experiences of women with mental illness and their opinion on routine screening for violence by nursing professionals in mental health care settings. Our findings showed that women with mental illness are experiencing various forms of domestic violence. Further, all the participants have expressed that nurses and other health care professionals (Psychiatrists, psychologists and social workers) should routinely enquire about violence experiences among women with mental illnesses.

The participants in this study were well aware of various forms of violence as they described the acts. For example, *hitting…pulling the hair, beating with a stick, torturing women, scolding women for simple things and purposefully making her cry,* etc. These findings were dissimilar to an Indian study conducted among psychiatric patients that found 42.7% of them were unaware of the word ‘domestic violence’. However, after explaining to them about the concept of domestic violence, almost all the participants (99.7%) admitted that they were experiencing violence in their lives.(16)

The participants in this study expressed that they are undergoing different forms of abuse. Most of the participants revealed violence by their husbands and other family members. They felt helpless because of their dependency on family members and the stigma associated with mental illness. Earlier studies supported these findings. A recent Indian study among persons with mental illness found that the prevalence of physical, emotional, sexual and economic violence was 16.3%, 25.3%, 2% and 11.3%, respectively. Younger age group and women were significantly associated with the occurrence of domestic violence.(16) Bhatia et al. (2016) reported that a majority (72%) of psychiatric outpatients were undergoing various forms of abuse. The most common form of violence was emotional (64%), followed by physical violence (39%) and sexual violence (21%).(12) A qualitative study from Bangladesh found that women with mental illness are experiencing all forms of violence (physical, emotional, sexual etc.). Most of the women were suffering from more than one form of violence. Sexual violence is a reality for a few women with mental illness but seldom is discussed.(11) These findings suggest the need to sensitize the family members about violence against women with mental illness.

Concerning nurses’ inquiry, most participants have expressed that nursing professionals did not inquire about violence experiences either on the day of admission or during their stay in the hospital. However, nurses have probed if they observe any scars or injuries on women’s bodies or if the patients are not having food or looking dull, not interacting with others, etc. They also expressed that nurses did not believe them even after they disclosed abuse and said they don’t have time to listen. Despite these experiences, most of them felt that disclosing violence helped them in ventilating their feelings. The present study's findings also underline the importance of mental health professionals in identifying and responding appropriately to disclosures of violence in mental health care settings.

The main barriers cited by the participants for disclosure of violence were; fear of consequences such as violence may be amplified and family members may not bring them for future follow-ups, felt that nurses are busy, feelings of helplessness i.e. believing that no one can improve her situation, nurses may make fun of them after listening to their experiences, nurses may inform family members, etc. These findings were similar to a study that explored barriers related to disclosure of violence to professionals were; fear of the consequences including fear of Social Services involvement and consequent child protection proceedings, fear that disclosure would not be believed, and fear that disclosure would lead to further violence; the hidden nature of the violence; actions of the perpetrator; and feelings of shame.(16) These findings could be attributed to stigma related to mental illness. Further lack of acknowledgement for the abusive experiences may provide limited opportunities to women. These results also highlight the need for mental health services to establish appropriate domestic violence strategies and responses to ensure optimal care for this vulnerable population.

In line with a qualitative meta-synthesis,(17) most of the participants favoured routine enquiry for domestic violence. Further, few participants recommended that staff receive training to improve their skills for routine screening.(17) The participants in this study expressed that a hospital is the best place to disclose their abusive experiences with health care professionals. They firmly believed that nurses might help them to find solutions. Hence, they felt routine screening might help them to feel that they are not discriminated. A study found that victimized women agreed that health care providers should screen female patients for domestic violence.(18) On probing, the participants expressed their expectations on the way they wanted to be enquired. All of the participants felt nurses should provide a supportive environment to disclose abusive experiences. For example, nurses should speak to them politely with respect and dignity. Hence, it is necessary to train nurses to identify violence among women with mental illness and respond to them in a non-judgmental and compassionate manner.

**Strengths and limitations.** To the best of our knowledge, this was the first qualitative study that explored abusive experiences among women with mental illness and their opinions on routine screening for domestic violence by nursing professionals in mental health care settings. The sample for this study was selected purposefully from different religious and cultural backgrounds. However, possible limitations of the present study include; sample bias as participants were recruited purposively and from a single setting. Further, acutely symptomatic women were excluded from this study. It may be quite possible that these women might be experiencing severe forms of domestic violence (physical and sexual violence). Hence, our findings may not be generalizable to all women with mental illness.

## Conclusion

The present study elicited abusive experiences among women with mental illness. The results indicate that women with mental illness were undergoing more than one form of violence and most of the participants supported routine screening by nursing professionals. Nurses play an important role in identifying and supporting abused women in mental health care settings. Therefore, it is crucial to explore nurses’ perceptions of routine screening of psychiatric patients for domestic violence. Further, it is necessary to enhance nurses’ knowledge and skills through appropriate educational interventions on domestic violence to provide holistic care to this vulnerable population.

Implications for psychiatric nursing practice. Based on these results, it is important to consider that nurses need to enquire routinely about abusive experiences of women with mental illness. Furthermore, nurses need to be adequately trained on how to enquire about violence and to provide appropriate interventions to victimized women. However, our findings may be useful in developing policies and guidelines related to routine screening for domestic violence in mental health care settings.
